# Strength and ability to implement the activities of daily living in elderly resident in rural areas

**Published:** 2016-09-30

**Authors:** Saulo Vasconcelos Rocha, Samara Souza dos Santos, Lélia Renata Carneiro Vasconcelos, Clarice Alves dos Santos

**Affiliations:** Departamento de Saúde II, Universidade Estadual do Sudoeste da Bahia, Itapetinga, Brasil

**Keywords:** Muscle strength, health services for the aged, rural population, aged, physical fitness, quality of life, social support

## Abstract

**Objective:**

To examine the association between muscle strength and the ability to perform basic and instrumental activities of daily living in elderly resident in rural areas of Jequie, Brazil.

**Methods:**

We performed a cross-sectional design study with a population of 104 individuals aged sixty or older, registered in the Family Health Unit of the district of Itajuru, Jequie-Brazil. Data collection was performed using a standardized instrument used as an interview, followed by the application of tests (bending arm with dumbbell and rising from a chair 30 sec). The basic and instrumental activities of daily living were investigated through the Katz and Lawton scales, respectively. The chi-square test with *p* ≤0.05 was used as a measure of statistical significance for bivariate analyzes between muscle strength and ability to perform daily activities.

**Results:**

The results showed a significant association between muscle strength and dynamic ability to perform activities of daily living.

**Conclusion:**

Reduced muscle strength is an important predictor of the functional ability of the elderly. Accordingly, it is recommended to observe muscle strength in actions directed at the elderly.

## Abstract (es)

**Objetivo:**

Analizar la asociación entre la fuerza muscular y la capacidad para la realización de las actividades básicas e instrumentales de la vida diaria en ancianos residentes de la zona rural del municipio de Jequie, Brasil.

**Métodos:**

Estudio de corte transversal, en una población de 104 individuos mayores a sesenta años, registrados en la Unidad de Salud de Familia del Distrito Itajurú, Jequié, Brasil. La recolección de los datos se realizó mediante una entrevista usando un instrumento estandarizado, después de la aplicación del test (brazo doblado con una pesa y levantarse de una silla en 30 seg. Las actividades básicas e instrumentales de la vida diaria fueron investigadas usando las escalas de Katz y Lawton, respectivamente. La significancia estadística para los análisis bivariados entre la fuerza muscular y la habilidad para realizar las actividades diarias se determinó usando la prueba de chi-cuadrado.

**Resultados:**

Se encontró una asociación significante entre la fuerza muscular y la habilidad dinámica para realizar las actividades de la vida diaria.

**Conclusión:**

La reducción de la fuerza muscular es un importante predictor de la habilidad funcional en los ancianos. De acuerdo a esto es importante se recomienda observar la fuerza muscular en las acciones dirigidas en los ancianos.

## I**ntroduction**

Population aging is a growing global phenomenon of great magnitude to public health because of the multiplicity of conditions and debilitating aspects that have caused important changes in the morbidity and mortality patterns, as well as in the health care of this population 1,2. 

Among the main changes associated with aging, there are the morphological and functional biological changes, mainly related to decreased skeletal muscle mass and muscle strength 3, which influence mainly the physical fitness of the elderly 4 and the ability to perform the activities of daily living (ADL), from the simplest, related to self-care, that involve the basic activities of daily living (BADL), to the most complex, such as instrumental activities of daily living (IADL), mainly related to the independence in the management of family life, use of domestic appliances, personal or public transport and control of their own medication and finance 5-7.

The decrease of the ability to perform the usual activities of daily living is more pronounced in older ages, especially among the elders above 85 years old 8. Besides, the biological factors 3, the demographic, socio-economic, cultural and psychosocial factors 9 play an important role in the decrease of the ability in the ADLs, with this in mind, particularities experienced in the social reality of the rural population should be considered, especially the difficulties of access to education, transportation, infrastructure of support, especially food and health care services 10. That disadvantage to access health care services among the elderly living in rural areas can mitigate the fragile condition due to age 11.

Considering the importance of muscle strength to maintain the capacity to perform the ADLs and the insufficiency of studies involving this topic in the elderly population living in rural areas, this study aims to analyze the association between muscle strength and the ability to perform basic and instrumental activities of daily living for the elderly living in rural areas.

## Materials and Methods

An epidemiological cross-sectional study was carried out, with a population of 104 individuals aged 60 years or more. A census was conducted with approximately 85% of elderly people registered in the Family Health Unit (FHU) in the Itajurú district, rural area of the municipality of Jequie, Bahia, Brazil.

The municipality of Jequie is located in the southwest region of the state of Bahia, with a territorial area of 3,035.42 Km^2^ and an estimated population of 151,820 inhabitants in 2010. In that municipality, the elderly population was of 16,323 representing approximately 10.5% of the total population. The municipality has 28 Family Health Teams (FHT), from which two are responsible for the coverage of the elderly residing in the rural area, and only the one located in the district of Itajuru accepted to participate in the study. 

The study excluded the individuals with amputation of the right upper limb, diagnosed with Alzheimer disease, dementia, or any other psychiatric or neurological disorder, which didn't allow the application of the questionnaire and tests. After the use of the exclusion criteria, the final population consisted of 95 elderly.

Data collection was carried out between the months of August and October 2011. A standardized instrument was used in the form of an interview, conducted in the household, followed by the application of tests conducted in the Family Health Units (FHU).

The collection instrument was constituted by questions about the socio-demographic characteristics (sex, age, marital status and education) and the elderly physical and functional ability, including evaluation of height, body mass, dynamic strength of the upper and lower limbs, and the assessment of the basic and instrumental abilities of daily living.

The nutritional status was verified by anthropometric measurements, measuring the body mass (measured standing barefoot with minimal clothing, using the Filizola weighing scale with capacity of 150 kg and sensitivity of 100 g); height (measured during exhalation using the anthropometer of the scale with the elderly standing, barefoot, using light clothing. The Body Mass Index (BMI) was calculated using the formula BMI = body weight (kg)/height^2^ (m).

To measure the strength of the upper limbs (MMSS) the arm bending test with halter was held, with the individual sitting in a chair with the feet flat on the floor and the dominant side of the body near the side edge of the chair seat, extended along the trunk and perpendicular to the ground, holding the weight (2 kg for women and 4 kg for men). From this position, the arm was bent towards the shoulder, returning to the fully extended position. At the command "start", the elderly performed the flexion and extension of the arm completely, keeping forearm still and performing the maximum of repetitions in 30 seconds 12. 

The strength of the lower limbs (MMII) was measured by a test consisting of getting up from a chair, which had a 43.18 cm seat and that was leaning against a wall to avoid slip. The participant was instructed to sit in the center of the seat with the back straight, fixed feet and crossed arms at the wrists against the chest. At the command "start", the elderly rose in full range of knee extension and hip, and returned to the sitting position. The individual was encouraged to get up and down as many times as possible in 30 seconds 12.

The values obtained in the strength tests (MMII and MMSS) were categorized according to the recommendations of Rikli and Jones 12.

The basic and instrumental activities of daily living were evaluated using the scales of Katz and Lawton, respectively. Katz scale evaluates the performance of an individual and the degree of needed assistance to perform basic activities of daily living (BADL's), such as bathing, getting dressed, going to the toilet, making transfers, urinary and fecal incontinence, and feeding 13. It was used as a cutoff point for functional incapacity with scores lower than 5 points.

To evaluate the instrumental activities of daily living (IADL's), it was used the version adapted in Brazil of Lawton and Brody scale 14, that evaluates the capacity of an individual to prepare meals, perform household chores, do the laundry, handle their own medication, use the telephone, manage money, go shopping and use public transportation. The cutoff point used to evaluate functional incapacity were scores lower than 17 points.

The statistical analysis was carried out with the statistical package SPSS (Statistical Package for the Social Sciences, version 13.0 for Windows). It was used the descriptive analysis of the studied variables (average, standard deviation, simple and relative frequencies). The square-chi Pearson (χ^2)^ with *p* ≤0.05 was used as a measure of statistical significance for bivariate analysis of muscle strength (MS) and the ability to perform daily activities.

The elderly who agreed to participate in the study signed the Consent Form, submitted and approved by the Research Ethics Committee of the State University of Southwest Bahia (CAE 0028.0.454.000-10), in compliance with the ethical principles of Resolution No. 196 of 10 October 1996 of the National Health Council (NHC) 15 . 

## Results

The average age of the studied population was 73.54 ± 9.43 years, and 57.9% of the subjects were female.

An average value of BMI of 25.58 ± 6.0 kg/m^2^ for the total sample was identified, 32.0% of the elderly evaluated were underweight/ eutrophic and 40.0% overweight. The women (27.18 ± 7.0 kg/m^2)^ had higher BMI than men (23.33 ± 3.49 kg/m^2)^.

More than half of respondents did not reach the recommended scores of muscle strength of upper and lower limbs (60.0% and 59.3%, respectively). The evaluation of BADL's showed that 96.6% of the elderly can perform their tasks independently, however, to perform IADL's only 16.0% are independent (Table 1).

**Table 1.** Values obtained from measurements of arm bending tests with halter and rising from a chair, and from the Katz and Lawton scales of elderly resident in rural areas, Jequié- BA 2011.

Scales	Variables	n	%
Arm Bending	Recommended	34	40.0
	Not recommended	51	60.0
Rising from a chair	Recommended	35	40.7
	Not recommended	51	59.3
Katz Scale	Independent	85	96.6
	Dependent	3	3.4
Lawton Scale	Independent	15	16.0
	Dependent	79	84.0

*Information losses: Arm bending (n= 10); Rising from a chair (n= 9); Katz Scale (n= 7); Lawton Scale(n= 1).

The muscle strength of the upper limbs was not associated with the ability to perform the BADL's with statistically significant levels (*p*= 0.401). However, when evaluating the association of upper limb strength with the capacity to perform the IADL's there was a statistical significance (*p*= 0.045) (Table 2).

**Table 2.** Association between upper and lower limbs strength and ability in carrying out the BADL's and IADL's in elderly resident in rural areas, Jequié- BA 2011.

	BADL's				IADL's			
Variable	Independent	Dependente	p value	Independente	Dependent	p value
Upper limbs strength*						
Recommended	32 (100)	0 (0.0)		2,401 (5.9)	32 (94.1)	
Not recommended	45 (97.8)	1 (2.2)	0.401	11 (22.0)	39 (78.0)	0.045
Lower limbs strength						
Recommended	32 (100)	0 (0.0)		2.727 (5.7)	33 (94.3)	
Not recommended	45 (97.8)	1 (2.2)	0.727	11 (22.0)	39 (78.0)	0.040

n(%)

*Information losses: BADL's (n= 17); IADL's (n= 11).

Findings similar to the upper limb strength were identified to evaluate the association between lower limb strength and functional ability, only statistically significant levels for the IADL's (*p*= 0.040) were observed (Table 2). 

## Discussion

Population aging is currently a reality in most societies. During the last thirty years, the number of elderly has grown significantly, a phenomenon that will continue to occur in the next years 1,2,16. This progressive increase in the elderly population occurs mainly due to the reduction in mortality levels. However, in developing countries, the impact of the increase in the number of elderly people to improve the living conditions of the population is still small, increasing the morbidity conditions, mainly related to chronic diseases 17, with increased demands for health services, setting up a problem of social and economic order for the care of this population 2,18.

The aging process does not occur homogeneously, and may be influenced by social, economic and cultural factors, interfering with the health conditions of the population 18, so residents in rural areas face significant precariousness of local structure of assistance and social support, especially with regard to health.

The inhabitants of these outlying areas are the most affected by the lack of health and social programs policies, since investments in health are uneven and sporadic 19. The same was observed by the Chinese government, where the elderly living in rural areas face difficulties to access health services, lack of doctors and medical facilities 20. 

In the present study a higher percentage of women were studied, a fact that confirms the estimates that indicate that in 2050, there will be 100 elderly women for 76 elderly men 21. This phenomenon may be due to the different mortality rate between the sexes, according to which men have a lower life expectancy than women. Another factor influencing the greatest number of older women in rural areas is male migration, which occurred in productive age to urban areas in search of better working conditions and income 22. 

In relation to muscle strength, superior upper limb-UL and lower member-LM, most of the elderly in the present study, didn't reach the minimum scores recommended by Rikli, Jones 12. The study carried out with 40 elderly women, aged between 60 and 70 years old, practitioners and non-practitioners of physical activity, shows that the average of repetitions of the arm bending exercise with dumbbell was lower than in the present study. In relation to the test of rising from a chair, the average of repetitions was similar 23.

The reduction of muscle strength can be explained by a reduced use of muscle usage along the aging process, causing a decrease in the levels of physical activity, as elderly people tend to remain seated and less active most of the time 24. 

Still, the physiological changes that occur during the aging process, the decrease of the functional ability in the medium and long term, mainly due to loss of muscle mass 25, makes the elderly more susceptible to fragility and to be dependent from care, and this is what probably influences more significantly the quality of life of the individuals 3,5,7, whereas to perform daily tasks it becomes necessary to produce strength in different lengths and speeds that are determined by the mechanical properties of the muscles 26.

In the present study the muscle strength of the UL and LMcontributed positively to the increase of the capacity to perform IADL's. Morphological and neural changes that occur during aging lead to decreased muscle strength and consequently reduced functional ability 27. Still, hormonal changes, mainly because of the reduction of testosterone levels in men and greater amount of body fat in women are events that can increase elderly exposure to losses of muscle mass with consequent decrease in functionality 28.

The reduction in strength adversely affects the performance of ADL's over the years, being women the most affected, because they are more vulnerable to the intensity of the deleterious effects of aging 6.

Aspects related to women's lifestyle, as for example, spending much of their time with the obligations of taking care of their offspring and housework, limiting their sport and leisure activities, may increase the likelihood of reduced muscle strength and consequently reduce the ability to perform daily activities 6.

Despite the factors related to the reduction of muscle strength during the aging process, the stimulation of muscle fibers, through regular physical activity, has been linked to the reduction of the deleterious effects of aging, contributing to improve functional exercise ability and the quality of life of the elderly 26.

On the other hand, poor nutrition can be understood by protein-calorie deficiency that induces disorder in the reception of the necessary nutrients for health maintenance, observed, in the present study, in 32% of the elderly who have a BMI below the recommended levels. This interferes negatively in the maintenance of muscle strength as it affects the musculoskeletal system and thus energy reserves, making individuals more vulnerable to functional losses 29.

As this is a cross-sectional study, it is not possible to estimate causality, so it only determines the correlation between variables. Another limiting factor was the region of the studied population, as the studies found in the literature, most of them, do not investigate elderly people living in rural areas.

Moreover, information was lost about arm flexion tests (n= 10); rising up from a chair (n= 9); Katz scale (n= 7); Lawton Scale (n= 1); ABVD´s (n= 17); AIVD's (n= 10), because of impossibilities due to injuries in the upper or lower limbs (diagnostic or reported by the evaluated) in the case of arm flexion motor tests and sit and get up from a chair. With regard to information on Katz Scale (n= 7); Lawton Scale (n= 1); BADL's (n= 17); IADL's (n= 10) the losses were due to the resistance of the respondents to answer some questions of the scale and to errors in the questions by the interviewers.

However, the study investigated a large contingent of elderly people living in rural areas, population that has been little investigated in population studies, thus generating important information for better understanding of the studied event.

## Conclusion

In this study an association between the dynamic muscle strength and the ability to perform the basic and instrumental activities of daily life was found, however, it was only statistically significant for the IADL's.

Due to the reduction in muscle strength that occurs during aging, and its association with functional ability, the elderly are predisposed to greater reliance to carry out their daily tasks, therefore, the development of actions aimed at health care to the elderly who attended at the Family Health Strategy is recommended, including the monitoring of living habits for the maintenance and improvement of muscle strength of the studied population.
